# Influence of implant protrusion length on non-grafting osteotome sinus floor elevation with simultaneous implant: a 3- to 9-year retrospective study

**DOI:** 10.1186/s40729-021-00304-3

**Published:** 2021-03-25

**Authors:** Yi Yu, Qiming Jiang, Zhengchuan Zhang, Xiaolin Yu, Feilong Deng

**Affiliations:** 1 Hospital of Stomatology, Guanghua School of Stomatology, Sun Yat-sen University, Guangzhou, People’s Republic of China; 2 Guangdong Provincial Key Laboratory of Stomatology, Sun Yat-sen University, Guangzhou, People’s Republic of China

**Keywords:** Bone formation, Dental implant, Non-grafting, Osteotome sinus floor elevation, Posterior maxilla

## Abstract

**Background:**

This study analyzed the influence of implant protrusion length (IPL) on the possible factors that affect the long-term outcomes utilizing non-grafting osteotome sinus floor elevation (OSFE) with simultaneous implant placement, and to explore the optimal range of IPL.

**Materials and methods:**

A retrospective study design was adopted. The clinical and radiographic data of 105 implants in 65 patients were collected after 3–9 (mean 5.04) years follow-up. IPL was divided into three groups (group1, IPL<2mm; group2, 2mm≤IPL<4mm; group3, IPL≥4mm). Endo-sinus bone gain (ESBG), peri-implant marginal bone loss (MBL), bone to implant contact length (BICL), and percentage of ESBG (%ESBG) were used to evaluate non-grafting OSFE. A Kaplan-Meier analysis was performed to assess the cumulative survival rate. Multiple linear regression model was used to explore the relationship between the possible influence factors and ESBG. Analysis of variance (ANOVA) was applied to explore the correlation of IPL with ESBG, MBL, BICL, and %ESBG.

**Results:**

A total of 102 implants in 62 patients fulfilled the survival criteria, giving the cumulative survival rates of 96.4% and 94.1% for implant-based analysis and patient-based analysis, respectively. The mean ESBG, MBL, and BICL at the latest follow-up were 1.95±0.88 mm, 0.58±0.68 mm, and 5.51±1.47 mm. ESBG was found to be positively correlated to IPL. A significant decreased bone formation efficiency was found when IPL was over 4 mm (*P*=0.02).

**Conclusions:**

An optimal range of IPL within 4 mm was recommended for better long-term outcomes when applying non-grafting OSFE with simultaneous implant placement.

## Introduction

Implant-supported rehabilitation in posterior maxilla is one of the most challenging procedure due to the limited residual bone height and poor bone quality [1]. To obtain sufficient bone volume, sinus floor elevation techniques have been developed over decades [2]. Osteotome sinus floor elevation (OSFE) is more widely used since the surgical procedure is less invasive, the healing period is shorter, and postoperative discomfort is minimal [3, 4], compared with lateral sinus floor elevation (LSFE). With the development of the implant materials, implant design, and surgical technique, OSFE has been demonstrated to be highly predictable in long-term studies [3, 5–8]. Even in cases with an extremely atrophic posterior maxilla with a residual bone height (RBH) less than 5 mm, an over 90% implant survival rate has been extensively reported [9–12].

Although OSFE is worldwide applied in clinical practice today, a debate on whether it is necessary to use bone grafting materials after elevating the sinus membrane through OSFE technique still remains controversial. Autologous bone or different types of bone substitutes have been used in OSFE to maintain the space for better outcomes according to the previous studies [10, 12, 13]. However, many studies investigated that no significant differences in clinical and histological outcomes were found whether grafting material was used or not [9, 11, 14–17]. Researchers indicate that grafting material utilization was not a prerequisite in the sinus floor elevation surgery regarding the osteogenic capability of the bony walls and Schneiderian membrane as well as the maintenance of endo-sinus space around implants beneath the elevated membrane [18, 19].

Implant protrusion length (IPL), part of the implant length protruding into the sinus, is a critical factor related to the new bone formation in the maxillary sinus. Several studies [14, 20–23] have found that the IPL was positively correlated with the endo-sinus bone gain (ESBG) following non-grafting OSFE. The protruding implant was considered as the maintenance for endo-sinus space under the elevated membrane, which contributes to bone formation around implants [22]. Therefore, greater ESBG can be obtained with a longer IPL.

However, due to the limitation of the osteogenic capability and elasticity of the membrane [24], it can be hypothesized that above a certain height in sinus, bone would not fill the newly created space and only the risk of intraoperative sinus membrane perforation and postoperative complications would increase. This hypothesis has been confirmed by some experiments in dog models. Sul et al. [25] found the implants with a longer IPL were not fully covered with intact membrane and did not obtain greater ESBG than those with the IPL of 4 mm. Both Zhong et al. [26] and Elhamruni et al. [27] reported the apexes of the implants were exposed in sinus without membrane or bone coverage with the IPL over 3 mm.

On the other hand, the importance of implant success of OSFE is not exclusively related to ESBG but also need sufficient bone-to-implant contact (BIC), which represents an important factor for implant survival [28]. Therefore, it can be reasonably speculated that there is an optimal range of IPL for implants when planning a non-grafting OSFE technique, which can provide sufficient ESBG and BIC for implant stability with better implant survival rates and clinical outcomes.

To the best of our knowledge, no clinical study to date has thoroughly assessed the influence of IPL on non-grafting OSFE technique and explored the optimal range of IPL. Hence, the aim of the present study is to analyze the correlation between IPL and the possible factors that would affect the long-term outcomes of non-grafting OSFE technique, and to explore the optimal range of IPL for implants. This was done by (a) evaluating the cumulative implant survival rate, (b) measuring ESBG under the elevated membrane, (c) measuring the bone-to-implant contact length (BICL), and (d) analyzing the potential influence factors related to the bone remodeling.

## Material and methods

### Study design and patient selection

This retrospective study was approved by the Ethics Committee of Guanghua School of Stomatology, Hospital of Stomatology, Sun Yat-sen University, China (Approval No. ERC-[2016]-12). The study procedure was conducted in strict accordance with Helsinki Declaration revised in 2008. All patients in the study signed the informed consent and were treated at the Department of Oral Implantology, Guanghua School of Stomatology, Hospital of Stomatology, Sun Yat-sen University, China, from December 2010 to December 2016. All the methods applied in the study were complied with the STROBE guidelines.

### Inclusion criteria

The patients were selected based on the following inclusion criteria:

(i) Age ≥18 years
(ii) Good systemic health without any uncontrolled disease
(iii) Good oral hygiene
(iv) Teeth loss in the posterior maxilla for at least 3 months
(v) Received non-grafting OSFE technique with simultaneous implant placement
(vi) Signed informed consent and capable to comply with the study protocol

### Exclusion criteria

The patients were excluded based on the following criteria:

(i) Uncontrolled systemic diseases
(ii) Untreated oral disorders
(iii) Severe acute or chronic sinus disease
(iv) Previous implants placement or bone augmentation surgery at the surgical site
(v) Heavy smoker (≥20 cigarettes per day)
(vi) Drug or alcohol addiction [29]
(vii) Bruxism [30]
(viii) Pregnant or attempting to get pregnant at the time of screening

### Surgical and prosthetic procedures

The details of procedures have been described in our previous study [31]. Briefly, all patients received an appropriate treatment related to endodontic or periodontal disorders before surgery. Periapical radiograph (MInray, SoredexPalodex Group Oy, Finland) was taken to estimate the height of the bone crest. Additional cone-beam computed tomography (CBCT) (NewTom VGi, Italy) was also required if patients have extremely atrophic posterior maxillae. The surgery was performed under local anesthesia with 4% articaine. A midcrestal incision was used for flap elevation, and vertical or periosteal release incision was not applied. The preparation of the implant site was performed with drills and ended 1 mm from the sinus floor based on radiography examinations. After preparation, a set of osteotomes with various diameters were used to elevate sinus floor by tapping in vertical direction to create a “greenstick” fracture. Tapping should be done gently to minimize the risk of Schneiderian membrane perforation. The Valsalva maneuver was performed to reinsure the membrane intactness (nose blowing test) [32]. No grafting materials were used. Implants (4.3×8 mm, 4.3×10 mm, 5×10 mm, NobelReplace, Nobel Biocare, Sweden; 5×6 mm, 5×8 mm, Bicon, Boston, USA) were placed in the prepared sites without tapping. Flaps were sutured with polyglycolic acid 4/0 sutures (OPTIME, Peters Surgical, France). Periapical radiograph or CBCT was taken after surgery.

All patients received Cefradine 500 mg four times for 5 days as a preventive method for infection, together with analgesics if needed. Oral hygiene education was performed, including using chlorhexidine gargle 10 mL twice daily and no tooth-brushing around implant sites for 7 days. Sutures were removed 7 to 10 days after surgery. After a healing period of 3-6 months, the patients were asked to come back for second-stage surgery and restoration. Periapical radiographs were taken to examine whether there was any peri-implant bone radiolucency. Single crowns (SCs) and multiple-unit implant-supported fixed dental prostheses (FDPs) were applied for restoration.

### Follow-up examination

The patients were recalled for radiographic and clinical examinations every year after restoration. The clinical assessment of implants, prostheses, and peri-implant tissues was conducted. The patients experiencing implant loss or other complications were recorded. Periapical radiograph was taken to evaluate the endo-sinus bone gain (ESBG), peri-implant marginal bone loss (MBL), and bone to implant contact length (BICL).

### Outcome measurements

#### Implant survival

The implant survival was evaluated by the criteria proposed by Buser et al. [33]: (i) the absence of clinically detectable implant mobility, (ii) the absence of pain or any subjective sensation, (iii) the absence of recurrent peri-implant infection, and (iv) the absence of continuous radiolucency around the implant.

#### Radiographic assessment

**Fig. 1:**
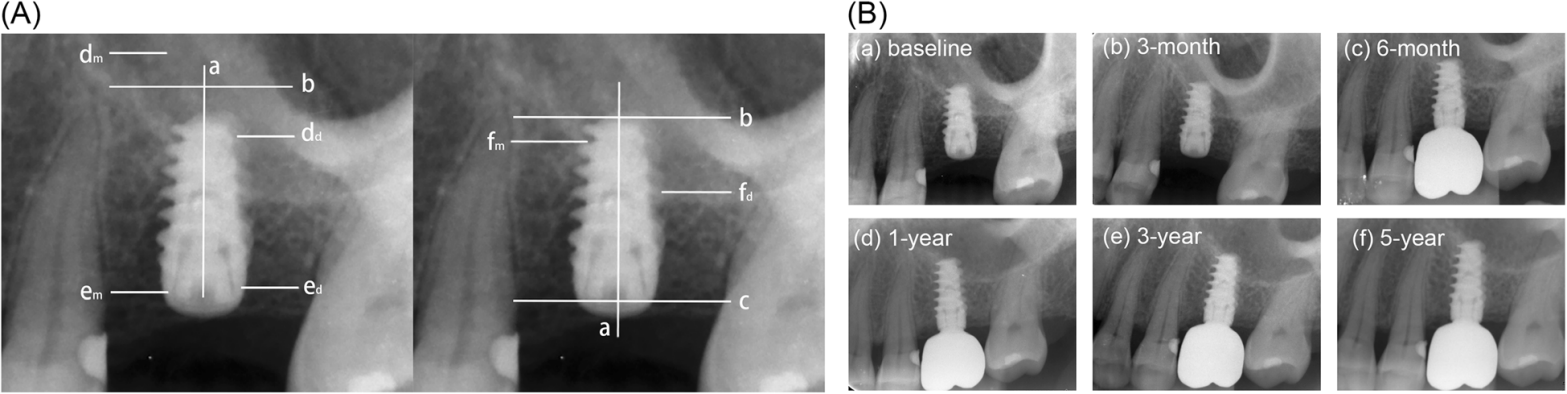
**a** Radiographic assessment. Reference lines were drawn as follows: (**a**) Implant longitudinal axis; (**b**) implant apex line: the most apical level of the implant, vertical to (**a**); (**c**) implant coronal line: the level of implant-to-abutment contact, vertical to (**a**); (**d**) Apical bone line: the most apical level of the new bone in the sinus, vertical to (**a**), at the mesial site (d_m_) and at the distal site (d_d_); (**e**) crest bone line: the most coronal level of bone-to-implant contact, vertical to (**a**), at the mesial site (e_m_) and at the distal site (e_d_); (**f)** Sinus floor line: the most coronal level of sinus floor cortical bone, vertical to (**a**) , at the mesial site (f_m_) and at the distal site (f_d_). **b** Endo-sinus bone remodeling after non-grafting OSFE

Digital radiographs taken at the baseline (immediately after surgeries) and every follow-up visit were assessed and analyzed by a radiography software program (Soderex, DigoraOptime, Finland). Some reference lines were drawn as Fig. 1 as follows:

(a) Implant longitudinal axis

(b) Implant apex line: the most apical level of the implant, vertical to (a)

(c) Implant coronal line: the level of implant-to-abutment contact, vertical to (a)

(d) Apical bone line: the most apical level of the new bone in the sinus, vertical to (a)

(e) Crest bone line: the most coronal level of bone-to-implant contact, vertical to (a)

(f) Sinus floor line: the most coronal level of sinus floor cortical bone, vertical to (a)

The following parameters were recorded at the mesial and distal sides for each implant, and then averaged:

• Implant length (IL): distance from (b) to (e)

• Residual bone height (RBH): distance from (e) to (f), assessed at the baseline

• Apical bone level (ABL): distance from (d) to (b), assessed at the baseline and every follow-up visit

• Peri-implant crestal bone level (CBL): distance from (c) to (e), assessed at baseline and every follow-up visit

• Bone to implant contact length (BICL): distance from (b) to (e). If the ABL is higher than the implant apex; distance from (d) to (e). If the ABL is lower than the implant apex, assessed at the baseline and every follow-up visit

• Implant protrusion length (IPL): distance from (b) to (f), assessed at the baseline; IPL was divided into three groups. Group 1, IPL<2mm; group 2, 2mm≤IPL<4mm; group 3, IPL≥4mm. Reasons were stated as follows:

The average IPL in some studies was around 2 mm and achieved a high percentage of ESBG [22, 23, 34]. Thus, we selected 2 mm as the first interval point in this study in order to see whether the %ESBG would be higher with a IPL over than 2 mm. Nedir et al. [21, 35] have proposed that grafting was unnecessary to achieve an average of ESBG around 4 mm. Therefore, 4 mm was chosen as the second interval point to see if more ESBG could be obtained in non-grafting OSFE when the IPL was more than 4 mm. The method of division was also consistent with some previous studies [14, 20, 34].

To account for the errors caused by radiographic distortion, all these measurements were adjusted to coefficient from the radio of “true implant length/implant length on radiograph.” Endo-sinus bone gain (ESBG) was the sum of IPL at baseline and ABL at each follow-up visit. If the newly formed bone is higher than the implant apex, ABL is positive; otherwise, it is negative; marginal bone loss (MBL) was measured by subtracting CBL at each follow-up visit from that at baseline; the value of the percentage of ESBG (%ESBG) was calculated by the formula of:

$$ \%\mathrm{ESBG}=\mathrm{ESBG}/\mathrm{IPL}\times 100\% $$

#### Statistical analysis

Data collection of all the radiographic measurements was carried out by two independent inspectors. If the difference between the two observed values was 0.5 mm or less, use the average value of these measurements, otherwise, rechecked radiographs and sought consensus. SPSS Software (SPSS 19.0; SPSS Inc., Chicago, IL, USA) was applied to perform the statistical analysis.

Descriptive statistics were performed in the study. Continuous variables were described with mean ± standard deviation (SD). Both absolute and relative frequencies distributions were provided for the qualitative variables. Kaplan–Meier survival curves were used to estimate implant survival over time. Due to the fact that the measurements of multiple implants within one patient might have correlation in nature, the implant survival rate was assessed by patient-based analysis and implant-based analysis, respectively. Multiple linear regression model was used to explore the relationship between the possible influence factors and ESBG at the latest follow-up. Analysis of variance (ANOVA) was used to analyze the correlation of ESBG, MBL, BICL, and the percentage of ESBG within different IPL groups. The homogeneity of variances and the linearity of the relationship between dependent and independent variables were tested. All *P* values were interpreted in a descriptive sense and have no confirmatory value. A *P* value smaller than 0.05 is considered statistically significant.

## Results

### Patient and implant information

A total of 105 implants placed simultaneous with non-grafting OSFE in 65 patients (37 males and 28 females) with a mean age of 51.2 (ranging from 23 to 77) years old were enrolled in the present study. The mean follow-up time was 5.04 years. Sixty-two implants with a length of 6 mm, 16 implants with a length of 8 mm, and 27 implants with a length of 10 mm were used. Ninety-two of the 105 implants were 5 mm in diameter and the rest of them were 4.3 mm. The characters and distribution of patients and implants were shown in Table 1.

**Table 1:** Patient and implant characters and distributions

	Gender			Age				Periodontal status						Smoking status		
	Male	Female		20-40	41-60		61-80	Treated periodontitis			Non-periodontitis			Smoking	Non-smoking	
Patient-based statistics	37	28		11	41		13	20			45			14	51	
	Implant length (mm)			Implant diameter (mm)		Implant site		RBH (mm)		IPL (mm)			Prosthesis		Implant system	
	6	8	10	4.3	5	Premolar	Molar	<5	≥5	<2	2-4	≥4	Single crown	Multiple-unit FDP	Nobel	Bicon
Implant-based statistics	62	16	27	13	92	10	95	65	40	32	58	15	88	17	39	66

Abbreviation: *RBH* Residual bone height; *IPL* Implant protrusion length; *FDP* Fixed dental prosthesis

### Baseline/radiographic measurements

The IPL and RBH of the implants were measured at the baseline. The IPL of the implants was ranged from 0.8 mm to 6.1 mm, with a mean value of 2.64±1.10mm. Thirty-two implants in group1 (mean: 1.44±0.33 mm, ranging from 0.8 to 1.9 mm), 58 implants in group 2 (mean: 2.88±0.54 mm, ranging from 2.0 to 3.9 mm) and 15 implants in group 3 (mean: 4.54±0.54mm, ranging from 4.0 to 6.1 mm) were measured. The average RBH was 4.69±1.60 mm (ranging from 2.2 to 8.6 mm). Sixty-five of the 105 implants were placed in sites with a bone height of less than 5 mm (mean: 3.63±0.73mm, ranging from 2.2 to 4.9 mm), and the other implants were placed in sites with RBH≥5mm (mean: 6.40±1.02mm, ranging from 5.0 to 8.6 mm).

### Implant survival rate

**Fig. 2:**
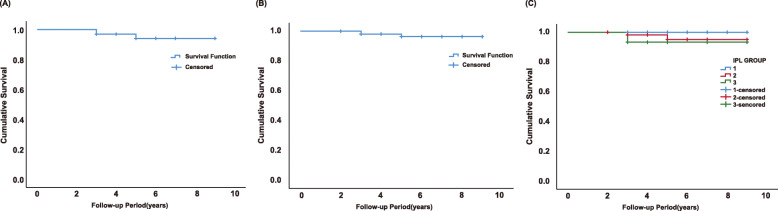
(**a**) Patient-based and (**b**) implant-based Kaplan-Meier curve indicating favorable cumulative survival rates for non-grafting OSFE. (**c**) Kaplan-Meier analysis of implants with different IPL groups at the baseline. High implant survival rates were shown and no significant difference was found between the three IPL groups (*P*=0.373)

Three implants in three patients failed during the follow-up period, so they were excluded from the study. The Kaplan-Meier analysis indicated a cumulative survival rate of 94.1% by patient-based analysis and 96.4% by implant-based analysis (100% for group1; 95.2% for group 2; 93.3% for group 3) and no significant differences were found between the three IPL groups (*P*=0.373). The results are shown in Fig. 2.

### Radiographic measurements of bone remodeling and influence factors

The mean ESBG, MBL, and BICL of the surviving implants at the latest follow-up were 1.95±0.88 mm, 0.58±0.68 mm, and 5.51±1.47 mm respectively. Multiple linear regression model detected a positive correlation between IPL and ESBG while no other factors included were found correlated with ESBG (Table 2). ESBG, MBL, and BICL in different IPL groups were also analyzed (Table 3). Significant differences were found between ESBG of each IPL groups while no statistically differences were detected between either MBL or BICL of each group. %ESBG in different IPL groups are shown in Table 4. The results showed a lower percentage of ESBG for group 3 when comparing with group2 (*P*=0.02).

**Table 2:** Multiple linear regression model for factors influencing ESBG

Parameter	Unstandardized coefficients		Standardized coefficients	*t*	Sig.
	*B*	Std. error	Beta		
Intercept	−0.240	0.585		−0.411	0.682
Factors					
IPL	0.692	0.061	0.860	11.367	0.000^*^
RBH	0.008	0.060	0.015	0.139	0.889
Follow-up period	−0.016	0.026	−0.036	−0.587	0.558
Covariables					
Gender					
Female	−0.086	0.123	-0.049	−0.696	0.488
Male	0	0			
Age					
61-80	0.353	0.179	0.160	1.969	0.052
41-60	0.191	0.144	0.105	1.322	0.189
21-40	0	0			
Periodontal status					
Treated periodontitis	0.057	0.142	0.030	0.398	0.691
Non-periodontitis	0	0			
Smoking status					
Smoking	−0.052	0.148	−0.025	−0.352	0.725
Non-smoking	0	0			
Implant site					
Molar	0.237	0.188	0.073	1.259	0.211
Premolar	0	0			
Implant length					
10 mm	0.779	0.460	0.386	1.694	0.094
8 mm	0.439	0.308	0.179	1.426	0.157
6 mm	0.	0			
Implant width					
4.3 mm	−0.191	0.354	−0.030	−0.539	0.591
5.0 mm	0	0			
Implant system					
Nobel	−0.009	0.188	−0.005	−0.050	0.960
Bicon					
Prosthesis type					
Multiple-unit FDP	−0.165	0.158	−0.068	−1.043	0.300
Single crown					

*Significant. *P*<0.05

**Table 3:** Mean ESBG, MBL, and BICL at the last follow-up in IPL groups

	Group 1	Group 2	Group 3
ESBG (mm)	1.05±0.33^*bc*^	2.21±0.62^*ac*^	2.97±0.84^*ab*^
MBL (mm)	0.61±0.78	0.59±0.68	0.51±0.34
BICL (mm)	5.35±1.11	5.69±1.70	5.12±1.19

*P* value calculated by the one-way ANOVA

The characters *a*,*b*,*c* indicated significant difference found when comparing to groups 1, 2, and 3 respectively under Games-Howell test (overall *P* value<0.05)

**Table 4:** The ESBG percentage distribution in different IPL groups and mean %ESBG

	Percentage of ESBG				Mean %ESBG (%)
	≥80%	60-80%	40-60%	20-40%	
Group1	11	15	6	0	72.0±15.1
Group2	32	15	9	0	77.0±14.5^*c*^
Group3	2	8	2	2	66.3±19.0^*b*^

*P* value calculated by the one-way ANOVA

The characters *b*, *c* indicated significant difference found when comparing to groups 2 and 3 respectively under Student-Newman-Keuls correction (overall *P* value<0.05)

## Discussion

The aim of this clinical study was to analyze the influence of IPL on long-term clinical outcomes and explore the correlation of IPL with ESBG, MBL, BICL, and %ESBG in non-grafting OSFE technique. In the present study, the 9-year cumulative implant survival rate of 96.4% was detected. The result revealed a favorable long-term prognosis of non-grafting OSFE with simultaneous implant placement which is slightly higher than Si et al. [23] and Rammelsberg et al. [8] who reported a 9-year survival rate of 90.6% and a 10-year survival rate of 93.7% respectively. The possible reason is that no implants were found lost during the healing period after surgery (early failure). Besides, the cumulative survival rates of different IPL groups were also calculated respectively, and no significant differences were found between groups in this study.

Our study found out a strong positive correlation between IPL and ESBG (*p=*0.000). No other factors included in multivariate analysis such as RBH, implant diameter and length, or patients’ features were found significantly relevant. Although other studies also reported similar results [14, 20, 23], no study revealed an appropriate range of IPL within which ideal long-term clinical outcomes can be achieved through non-grafting osteotome sinus floor elevation. In the present study, apexes of some implants were not covered by newly formed bone at the last follow up. Jensen et al. reported that the endo-sinus bone formation did not always reach the apex of the implant, which was conflicted with an old view that long-term success of implants can only be guaranteed with the implant fully embedded in bone [36]. Caban et al. [37] noted a bone resorption from the apex of implants at 10-year follow-up and regarded this resorption as a physiological process which did no harm to the implant stability. Nedir et al. [5] confirmed this opinion, reporting a 10-year implant survival rate of 100%. These results suggested that a fully embedded implant tip may no longer be a prerequisite for a favorable long-term prognosis.

Although the poor osteogenesis found near the implant tips do no harm to the long-term prognosis, an excessive IPL was related to an increased risk of membrane perforation [38], and may not help endo-sinus bone formation. Histological evidences revealed a limited osteogenic capability of non-grafting osteotome sinus floor elevation. Besides, some researchers also detected that the apexes of implants were not fully covered with newly formed bone when the IPL was over 3 mm in dog models [26, 27].

A significant decrease in the percentage of ESBG was found in this study when IPL was higher than 4 mm, indicating a decreased bone formation efficiency. The result suggests that an IPL over 4 mm may not be necessary for non-grafting osteotome sinus floor elevation as the ESBG hardly reach the excepted bone height. This may be owing to two following reasons: the limited osteogenic potential of the Schneiderian membrane and the collapse of the tented space.

Bone formation efficiency around the apex of the implant may be affected by the uncertain osteogenic capacity of the Schneiderian membrane. Different from the grafting sinus floor elevation technique which depends on the remodeling of graft materials, the mechanism of bone formation in non-grafting sinus floor elevation stays controversial. Multipotent mesenchymal stem cells were derived from Schneiderian membrane which indicated an osteogenic capability of the maxillary sinus membrane [18, 39]. This result was verified in studies which showed a gradual new bone formation and up to 86.5% implant tips were embedded in the new bone in non-grafting sinus floor elevation within the 1-6 years follow-up [23]. However, the result of the present study doubted this hypothesis as the bone formation around the implant tip was below expectation which is also detected by Nedir et al. [5]. Experimental studies in animal models also indicated a questionable or weakened osteogenic potential of the maxillary sinus membrane. Rong et al. [19] raised the sinus floor in dogs shielding either the bony walls or the Schneiderian membrane. New bone formation was detected in both groups whereas the bone formation in the bone shielding group was limited near the sinus membrane. They concluded that Schneiderian membrane did show osteogenic capacity while the potential is much weaker than that of the surrounding bony walls. In case of non-grafting sinus augmentation, whether the Schneiderian membrane is still involved in the endo-sinus bone formation remains unclear considering the situation that no graft material can serve as a scaffold for stem cell migration. Scala et al. [40] carried out non-grafting sinus floor elevation with simultaneous implant placement in monkeys. Histologic observations revealed that the new bone formation originated from the sinus floor and extended toward the apex of implants without the influence of Schneiderian membrane. They also found out that the bone formation failed to exceed 4.5 mm over the sinus floor which is consistent with the present study. The results implied that the Schneiderian membrane may do a weaker contribution to endo-sinus bone formation of non-grafting OFSE leading to a decreased osteogenic efficiency around the apex of implants.

The collapse of Schneiderian membrane may serve as another factor limiting the osteogenic capability of non-grafting OFSE, since space maintaining was considered to be playing a crucial role in sinus augmentation. With the increased IPL, the space around the apex of the implants created by the lifted Schneiderian membrane tends to collapse as Scala et al. [40, 41] reported in animal studies. Histologic assessments showed Schneiderian membrane lining the implant surface at the apex of implants. In another experimental study, Xu et al. [42] reported an increased number of osteoclasts beneath the Schneiderian membrane at the non-grafting side compared with grafted side indicating a progressive re-pneumatization of the sinus. Clinical studies reported similar results. A recent randomized clinical trial pointed out that the tenting stability by implants alone was limited and insufficient to maintain the created space after sinus augmentation with excessive IPL when comparing grafting and non-grafting sinus augmentation with simultaneous implant placement [43]. The Schneiderian membrane with an increased membrane tension was more likely to collapse and the tension of the membrane might also transfer a compression force to the bone thus activating the bone resorption procedure [44]. Thus, it can be inferred that the limited space maintaining capacity of the implants also restrained the osteogenesis for non-grafting OFSE. The IPL of this technique needs to be restricted in order to avoid excessive tension to the Schneiderian membrane.

Besides, the present study also found out that there was no statistical difference in BICL among three IPL groups. The average BICL at the last follow-up which represents a stable bone anchorage supporting the implant was 5.51±1.47 mm. Despite this limited bone anchorage, a favorable accumulated survival rate was still guaranteed. This may be benefited from the bicortical engagement as our previous study [45] reported a 100% survival rate for implants with bicortical-fixed technique. The result indicated that considering the decreased bone formation efficiency and the limited BICL obtained, the placement of longer implants with an excessive IPL over 4 mm may not be necessary.

The level of MBL serves as another important monitoring indicator to evaluate the stability of the long-term clinical outcome of implants [46]. The mean MBL in this present study was 0.58±0.68 mm at the last follow-up which is consistent with other long-term researches [5, 23, 37]. Four implants in three patients with severe MBL>2mm were detected. It should be noted that all these implants with severe MBL were splinted and suffering from poor oral hygiene. However, no significant differences were found between MBL of splinted or non-splinted implants. This result is supported by the previous studies [47–49]. Batista et al. [47] reviewed 19 studies including 4215 implants in 2185 patients and quantitative analysis found no significant difference for MBL between splinted and non-splinted implants. Despite this, they still pointed out that these favorable results could be due to the fact that individuals participating in these clinical trials followed an adequate maintenance protocol. Thus, greater importance should be given to appropriate oral hygiene and adequate maintenance protocol for patients, especially those with splinted restorations.

RBH was considered to be another crucial factor affecting the clinical outcome of non-grafting osteotome sinus floor elevation. Fabbro et al. [10] reviewed 19 articles and reported an overall survival rate of 92.7% for 331 implants with RBH<5 mm and 96.9% for 2525 implants with RBH>5 mm, suggesting a more favorable prognosis when RBH is over 5 mm. While, on the other hand, Rammelsberg et al. [8] reported a satisfactory 95.7% 10-year survival rate for implants placed in RBH of 4–6 mm, and 77.4% for those extreme cases with RBH of merely 1-3 mm. They suggested that non-grafting osteotome sinus floor elevation can still be considered even for these extreme cases as a minimally invasive treatment option. In the present study, 65 out of 105 implants were placed in RBH<5 mm (mean: 3.63±0.73 mm, ranging from 2.2 mm to 4.9 mm) and a remarkable 9-year cumulative implant survival rate of 96.4% was achieved indicating that non-grafting osteotome sinus floor elevation can serve as a promising minimally invasive treatment approach for cases with RBH<5 mm and might still be carefully considered even for cases with extremely compromised RBH less than 3 mm.

## Conclusions

The present study reported a strong positive correlation between IPL and ESBG. However, an IPL over 4 mm may not be necessary for non-grafting osteotome sinus floor elevation as the bone formation efficiency (ESBG%) reduced. Therefore, an optimal range of IPL within 4 mm was recommended for better long-term outcomes when applying non-grafting OSFE technique with simultaneous implant placement.

## Data Availability

The datasets used and analyzed during the current study are available from the corresponding author on reasonable request.
